# DANet: Joint Density- and Semantics-Adaptive Convolution for 3D Point-Cloud Semantic Segmentation

**DOI:** 10.3390/s26144561

**Published:** 2026-07-18

**Authors:** Weijian Hu, Shuning Wang, Lingfang Li, Jikai Zhang, Ke Han

**Affiliations:** 1 School of Transportation and Logistics, Southwest Jiaotong University, Xian Road, 999, Chengdu 611730, China; huweijian@imust.edu.cn (W.H.);; 2 School of Digital and Intelligent Industry, Inner Mongolia University of Science and Technology, 7 Aerding Street, Baotou 014010, China; wangshuning1999@stu.imust.edu.cn (S.W.); jkzhang0314@imust.edu.cn (J.Z.)

**Keywords:** 3D point-cloud semantic segmentation, LiDAR, depth sensing, adaptive convolution, non-uniform sampling, 3D perception

## Abstract

Semantic segmentation of 3D point clouds remains difficult when LiDAR or depth-camera data are sampled unevenly. This paper presents DANet, a 3D semantic segmentation framework built on joint density- and semantics-adaptive convolution. Its core operator, Density-Adaptive Radius Convolution (DAR-Conv), predicts point-wise neighborhood radii before feature aggregation by combining density-driven initialization with semantics-aware modulation. In this way, dense regions can use compact receptive fields, whereas sparse or semantically complex regions can draw on broader contextual support. DANet also includes a Gated Adaptive Cross-Layer Fusion (GACF) module, which aligns encoder–decoder features and performs gated fusion with residual refinement. Experiments on S3DIS and NPM3D show that DANet obtains the highest reported mean accuracy (mAcc) among the compared methods on S3DIS, and high mean Intersection over Union (mIoU) and overall accuracy (OA) on NPM3D, supporting the usefulness of density- and semantics-aware receptive-field adaptation.

## 1. Introduction

Point-cloud semantic segmentation aims to assign a semantic label to each point in irregular 3D data and is a central task in 3D scene understanding. It supports applications such as autonomous perception, urban sensing, robotic navigation, and environmental reconstruction. As LiDAR and depth-camera systems become more widely used, large-scale point clouds acquired in practical environments are increasingly available. This has made accurate and efficient semantic interpretation of complex 3D scenes an active research topic [1,2,3].

Accurate segmentation remains challenging because point clouds are unordered, irregular, and often non-uniformly sampled. Recent studies show that non-uniformity, sparsity, and structural complexity create persistent difficulties for feature learning and model generalization [1,4,5]. These difficulties are especially evident in LiDAR and depth-camera point clouds, where sampling patterns vary with acquisition geometry, sensing distance, viewing angle, occlusion, and scene composition. The resulting local neighborhoods may be inconsistent across a scene, which weakens geometric representation and boundary discrimination.

Existing studies have improved both local feature aggregation and global contextual modeling. Enhanced neighborhood representation, enlarged receptive fields, and long-range dependency modeling have all led to notable gains in segmentation performance [4,5,6]. Even so, it is still difficult to balance fine-grained geometric detail and contextual completeness under density-varying conditions. If neighborhood construction does not adapt to spatial density and semantic complexity, aggregation may become redundant in dense regions and insufficient in sparse regions. Current adaptive designs, including deformable point convolutions, dynamic kernel assembly, adaptive sampling, and attention-weighted aggregation, improve different parts of local representation learning. However, they often adapt aggregation weights, kernel responses, or sampled points only after neighborhood support has been defined. This motivates a mechanism that makes the receptive-field extent itself responsive to both density and semantics.

Based on this motivation, we propose DANet, a 3D point-cloud semantic segmentation framework based on joint density- and semantics-adaptive convolution. DANet integrates variable-radius prediction, KPConv-based aggregation, and semantic modulation into a radius-adaptive local operator. A density-aware branch estimates the required geometric support, a semantic-guidance branch modulates this support according to feature relationships, and KPConv is applied over the resulting point-specific neighborhoods. This design allows neighborhood modeling to respond to both local density variation and semantic structure, reducing sensitivity to uneven sampling in LiDAR and depth-camera data. We also introduce an adaptive cross-level fusion strategy to strengthen interactions between geometric details and high-level semantics. The network is trained with a joint objective that combines segmentation supervision, geometric consistency regularization, and parameter regularization, with the aim of stabilizing training and improving boundary discrimination. Together, these components help DANet balance local detail preservation and global contextual understanding across indoor and outdoor scenes.

The main contributions of this work are summarized as follows:

- We propose DANet, a 3D point-cloud semantic segmentation framework designed to handle non-uniform point distributions commonly encountered in 3D acquisitions.
- We propose DAR-Conv, a joint density- and semantics-adaptive convolution operator that differs from prior adaptive aggregation methods by adapting the neighborhood radius before feature aggregation rather than only adjusting aggregation weights or deforming kernels within a fixed support.
- We design GACF, a gated adaptive cross-layer fusion module that enhances the interaction between geometric and semantic features through feature alignment, gated fusion, and residual refinement.
- We conduct extensive experiments on S3DIS and NPM3D, showing the highest reported mAcc among the compared methods on S3DIS and high mIoU/OA on NPM3D across indoor scans and outdoor surveys.

## 2. Related Work

### 2.1. 3D Point-Cloud Semantic Segmentation

Point-cloud semantic segmentation has attracted considerable attention due to advances in deep learning for 3D data. Existing methods are generally categorized into projection-based, voxel-based, and point-based approaches, depending on how point clouds are represented and processed.

Projection-based methods convert 3D point clouds into 2D representations, such as range images or multi-view projections, enabling the use of mature convolutional neural networks originally developed for images. Although these approaches are computationally efficient, they often suffer from geometric information loss and limited spatial consistency due to the projection process [7,8]. Voxel-based methods discretize point clouds into regular grids and apply 3D convolutions for structured feature learning. While they facilitate large-scale context modeling, they introduce a trade-off between spatial resolution and computational cost, especially for high-resolution scenes [9,10].

Point-based methods directly operate on raw point sets and thus better preserve geometric fidelity. PointNet++ introduced hierarchical feature learning on point sets through local neighborhood grouping and has become a foundational framework in this field [11]. Subsequent work has improved local aggregation and scalability, including KPConv, which employs deformable kernel-point convolution for flexible geometric modeling [12], and RandLA-Net, which enables efficient large-scale point-cloud segmentation through random sampling and local feature aggregation [13]. Graph-based methods, such as Superpoint Graphs, improve large-scale semantic segmentation by grouping points into geometrically coherent superpoints and reasoning over graph structures [14]. More recently, transformer-based architectures, such as Point Transformer [15], PointNeXt [16], and Point Transformer V3 [17], have significantly strengthened contextual modeling by capturing long-range dependencies through attention mechanisms. Recent studies have further improved local-context propagation and structure-aware representation learning, such as LCPFormer, SCF-Net, and adaptive spatial graph transformers [3,18,19]. These advances further highlight the importance of stable local–global interaction under irregular density and complex geometry. Despite these advances, the performance of point-based methods still depends heavily on neighborhood construction and receptive-field design, particularly under irregular and density-varying conditions.

### 2.2. Adaptive Neighborhood and Receptive-Field Learning

Local neighborhood construction plays a crucial role in point-cloud feature learning because it determines the spatial support for both geometric representation and semantic aggregation. Traditional approaches typically rely on fixed neighborhood definitions, such as k-nearest neighbors or fixed-radius search. Although simple and efficient, these strategies lack flexibility when handling non-uniform point distributions [11,13].

To handle irregular sampling, several studies have explored adaptive mechanisms for local feature aggregation. PointNet++ uses hierarchical grouping and multi-scale neighborhoods to alleviate non-uniform sampling effects [11], and PointASNL combines adaptive sampling with non-local modeling [20]. KPConv introduces deformable kernel points to improve flexibility in local geometric modeling [12], while PAConv dynamically assembles convolution kernels to better adapt to local structures [21]. In addition, attention-based methods, such as Point Transformer and its variants [15,17,22,23], enable adaptive feature weighting based on spatial and semantic relationships, thereby strengthening contextual interaction. These approaches have demonstrated strong performance gains in both local representation and global context modeling.

Although these methods are closely related to DAR-Conv, their adaptation targets are different. Multi-scale grouping and adaptive sampling mainly decide which local samples are used; deformable convolution adjusts kernel locations or kernel responses; dynamic-kernel methods assemble convolution weights according to local geometry; and transformer-style modules primarily learn attention weights over a constructed neighborhood. In contrast, DAR-Conv focuses on the support region before feature aggregation by predicting a point-wise convolution radius from two complementary cues: local point density and semantic response. The resulting radius determines the neighborhood on which KPConv is applied, so KPConv acts as the aggregation backbone rather than the source of the proposed adaptivity. This distinction enables DAR-Conv to reduce redundant aggregation in dense areas while expanding context in sparse or semantically complex regions.

However, most existing methods focus on adaptive feature aggregation within predefined neighborhoods, while neighborhood extent is still partially constrained by fixed rules, manually selected scales, or heuristic designs. As a result, these methods may struggle to simultaneously handle dense regions with redundant information and sparse regions with insufficient context. More importantly, existing approaches rarely consider the joint influence of local density variation and semantic structure when determining receptive fields. This limitation motivates the development of a more flexible mechanism that can dynamically adjust neighborhood support based on both geometric distribution and semantic cues.

### 2.3. Summary and Motivation

Existing point-cloud semantic segmentation methods have achieved substantial progress through improved feature aggregation, adaptive weighting, and contextual modeling. Density variation, however, remains a fundamental challenge. Most methods still do not explicitly integrate density-aware and semantics-aware information into a unified framework for adaptive receptive-field learning. DAR-Conv is therefore complementary to prior adaptive convolution and attention mechanisms: it learns when to shrink or enlarge the neighborhood support, while using KPConv only after this support has been determined.

To address this limitation, DANet introduces a joint density- and semantics-adaptive convolution mechanism. Its local receptive fields are adjusted according to both point density and semantic responses, with the goal of improving representation quality under non-uniform sampling conditions.

## 3. Methodology

### 3.1. Overall Architecture of DANet

**Figure 1:**
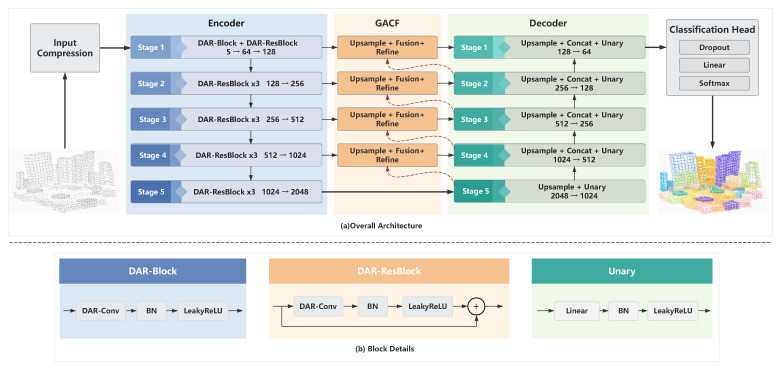
Overview of the DANet architecture.

DANet is a hierarchical encoder–decoder network for 3D point-cloud semantic segmentation, and its overall architecture is shown in Figure 1. The framework consists of an input compression module, an encoder, a GACF module, a decoder, and a classification head.

At the input compression module, voxel-grid subsampling is applied to reduce point density and computational overhead in large-scale 3D scenes. This operation changes point-set cardinality while preserving the representation format of each retained point. By suppressing redundant local samples, the compression step also improves the stability of subsequent neighborhood feature extraction.

The encoder contains five hierarchical stages. Stage 1 performs initial local encoding with a DAR-Block followed by a DAR-ResBlock, with channel progression from 5 to 64 and then to 128. Here, DAR-Block denotes a basic local encoding block built around a DAR-Conv operator, while DAR-ResBlock denotes its residual variant, which combines DAR-Conv-based feature extraction with a shortcut connection for deeper hierarchical learning. Stages 2–5 each employ three DAR-ResBlocks, progressively increasing feature channels from 128 to 256, 512, 1024, and 2048 while reducing spatial resolution. This design enlarges the receptive field and gradually shifts the representation focus from fine-grained geometry to high-level semantics. DAR-Conv serves as the core local operator in these stages to reduce sensitivity to non-uniform point distributions.

The decoder follows a progressive top-down process. Starting from the deepest encoded representation, features are upsampled stage by stage and fused with encoder features at corresponding resolutions through skip connections. In this process, GACF acts as the core cross-level fusion module: it aligns encoder–decoder features before fusion, adaptively gates their relative contributions, and refines the fused representation. As a result, the decoder recovers fine spatial details while preserving semantic consistency across scales.

After decoding, the refined point-wise features are fed into a classification head to produce per-point semantic predictions. DANet thus follows an information flow of compression, hierarchical encoding, cross-level adaptive fusion, progressive decoding, and point-wise classification, which is suited to structurally complex and density-varying 3D scenes acquired by different sensing devices.

### 3.2. Density-Adaptive Radius Convolution

**Figure 2:**
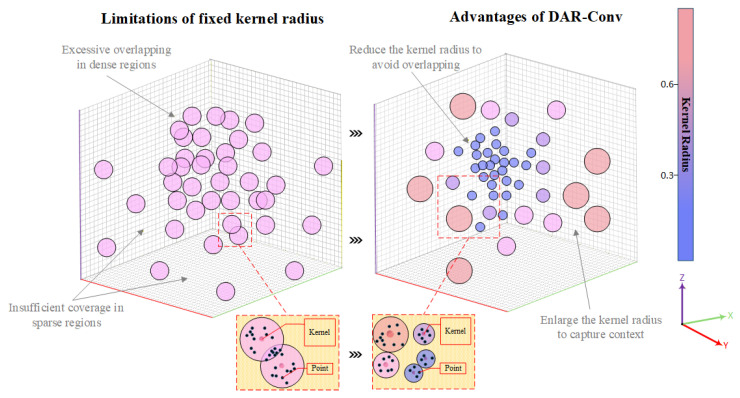
Limitations of fixed-radius neighborhoods and the adaptive receptive-field strategy in DAR-Conv.

Point clouds acquired by LiDAR, depth cameras, and other 3D sensing devices often exhibit pronounced spatial non-uniformity in sampling density. Variations in acquisition geometry, viewing direction, sensing distance, occlusion, and scene complexity can produce substantial density differences across local regions. Under such acquisition-dependent conditions, fixed-radius neighborhood modeling has two major limitations: redundant points may blur local geometric details in dense regions, whereas insufficient neighborhood support in sparse regions can lead to incomplete contextual representation and unstable semantic predictions (see Figure 2, left).

DAR-Conv adjusts neighborhood receptive fields according to local point distributions: neighborhoods are contracted in high-density regions to suppress redundancy and expanded in low-density regions to recover contextual information (see Figure 2, right). This behavior is useful for LiDAR and depth-camera point clouds, whose density patterns change markedly with sensing distance and visibility. On top of the density-aware adjustment, DAR-Conv uses semantic responses to modulate the radius-scale prediction, allowing the final receptive field to reflect both local density variation and semantic-structure complexity. The semantic modulation reduces the instability that can arise from density-only adjustment, especially near semantic boundaries and structural discontinuities. By adapting the radius before feature aggregation—through density-aware scale initialization and semantics-aware scale modulation—DAR-Conv produces more stable local representations under strongly non-uniform sampling and supplies higher-quality features for subsequent encoding and cross-level fusion.

**Figure 3:**
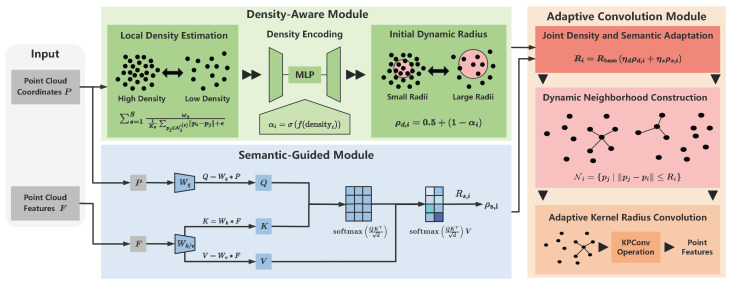
Architecture of DAR-Conv.

The DAR-Conv architecture, illustrated in Figure 3, consists of three components: a density-aware module, a semantic-guidance module, and an adaptive convolution module. Let the input point cloud be represented by spatial coordinates $P\in R^{N\times 3}$ and point features $F\in R^{N\times C}$. DAR-Conv processes these two types of information in separate branches and combines them in the final convolution. The density-aware module takes *P* as input and predicts an initial density-based radius-scale factor, providing geometry-level adaptation of the convolutional receptive field according to local point density. The semantic-guidance module takes both *P* and *F* as input and generates semantic modulation signals, which refine the radius near semantic boundaries and structurally complex regions. The adaptive convolution module then predicts the final radius from the density and semantic scale factors and constructs dynamic neighborhoods. Feature aggregation within these neighborhoods is performed using KPConv. In this form, DAR-Conv preserves the basic convolution computation while redefining neighborhoods adaptively, so the operation is better aligned with the spatial distribution and semantic structure of point clouds. The three components form a feature-extraction pipeline from local spatial-distribution perception and semantic-relationship modeling to dynamic-neighborhood convolution, improving feature quality in complex density-varying scenes.

This design is related to, but different from, existing adaptive point-cloud operators. In KPConv, deformation improves the flexibility of kernel-point locations, but the neighborhood support is still determined before aggregation [12]. In PAConv, dynamic kernel assembly changes convolution weights rather than explicitly predicting the point-wise support radius [21]. In transformer-based approaches, attention mainly reweights relationships among points within a constructed local or global token set [15,22]. By contrast, DAR-Conv uses density and semantic responses to adjust the local support before applying KPConv aggregation, which helps connect receptive-field selection with both sampling density and semantic structure.

#### 3.2.1. Density-Aware Module

The density-aware module generates an initial radius-scale factor from the local spatial distribution of points, providing geometric receptive-field adaptation for subsequent convolution. For each point $p_{i}$, multi-scale ball queries with *S* radii ${\left\{r_{s}\right\}}_{s=1}^{S}$ produce neighborhood sets $N_{i}^{\left(s\right)}$. We first compute a density estimate at each scale from the average neighbor distance and then fuse the multi-scale estimates:

(1) $${\mathrm{density}}_{i}^{\left(s\right)}=\frac{1}{\frac{1}{K_{s}}\sum_{p_{j}\in N_{i}^{\left(s\right)}}∥p_{i}-p_{j}∥+\epsilon },$$

(2) $${\mathrm{density}}_{i}=\sum_{s=1}^{S}\omega_{s}{\mathrm{density}}_{i}^{\left(s\right)},$$

where $K_{s}=\left|N_{i}^{\left(s\right)}\right|$ is the number of valid neighbors at scale *s*, ϵ is a small constant for numerical stability, and $\omega_{s}$ represents normalized scale-fusion weights satisfying $\omega_{s}\geq 0$ and $\sum_{s=1}^{S}\omega_{s}=1$. Under this definition, denser neighborhoods correspond to smaller average distances and thus larger density values, whereas sparser neighborhoods produce smaller density values. The fused density preserves local compactness information from multiple support radii, making it more suitable for non-uniform point clouds than a single-scale count-based statistic.

After obtaining local density, we encode it through a learnable mapping to generate a bounded density response. Specifically, a lightweight multilayer perceptron performs the following nonlinear mapping:

(3) $$\alpha_{i}=\sigma \left(f({\mathrm{density}}_{i})\right),$$

where f(·) denotes a learnable mapping and σ(·) is the sigmoid function that constrains the output to a bounded range. The density-based radius-scale factor is then computed with inverse scaling so that larger density responses reduce the radius scale:

(4) $$\rho_{d,i}=0.5+\left(1-\alpha_{i}\right),$$

where $\rho_{d,i}$ is a dimensionless density-based radius-scale factor. The physical radius is introduced later after density and semantic scale factors are fused.

Through this process, the density-aware module adaptively adjusts the receptive field according to local distribution patterns. In dense regions, the inverse radius scaling encourages compact neighborhoods and suppresses redundant neighbors, helping preserve local geometric details. In sparse regions, lower density responses produce a larger radius-scale factor, which can expand contextual support after scale fusion. Therefore, this module provides an initialization that better matches local geometry and improves feature modeling under non-uniform sampling.

#### 3.2.2. Semantic-Guidance Module

Although density-based modulation can partially mitigate non-uniform sampling effects, it is still insufficient for capturing semantic structures in complex scenes. This limitation is particularly evident near semantic boundaries or structural transition regions, where geometry-only modulation may produce unstable representations. To address this issue, we introduce a semantic-guidance module that provides complementary semantic cues for radius adjustment.

Unlike the density-aware module, which only uses coordinates, the semantic-guidance module takes both point coordinates $P\in R^{N\times 3}$ and point features $F\in R^{N\times C}$ as input. Coordinates describe local geometric relations, while features encode higher-level semantics. Jointly modeling both enables a more complete characterization of semantic relevance between points and their neighborhoods. We first project them into query(*Q*), key(*K*), and value(*V*) representations:

(5) $$Q=PW_{q},\;K=FW_{k},\;V=FW_{v},$$

where $W_{q}$, $W_{k}$, and $W_{v}$ are learnable projection matrices. After projection, coordinates provide geometric positional priors and features encode semantic similarity.

Based on these representations, we model point–neighborhood relations with attention. To avoid dense global N×N attention, this operation is computed only within the local sampled support $N_{i}^{s}$ of each center point:

(6) $$S_{i}=\mathrm{softmax}\left(\frac{Q_{i}K_{N_{i}^{\left(s\right)}}^{T}}{\sqrt{d}}\right),\;A_{i}=S_{i}V_{N_{i}^{\left(s\right)}},$$

where *d* is the feature dimension, and $K_{N_{i}^{s}}$ and $V_{N_{i}^{s}}$ denote the key and value features restricted to the local sampled support. Since radius modulation requires a scalar factor, we further map each point-wise attention response to a scalar semantic coefficient:

(7) $$R_{s,i}=\sigma \left(g(A_{i})\right),$$

where g(·) is a learnable projection (e.g., a one-layer MLP), and thus, $R_{s,i}\in \left(0,1\right)$. This response provides a compact summary of local semantic dependencies; in practice, it may vary more around class boundaries or structural changes and remain more consistent in semantically homogeneous regions. To avoid directly combining a semantic coefficient with a physical radius, we convert the semantic response into a dimensionless semantic radius-scale factor:

(8) $$\rho_{s,i}=0.5+R_{s,i},$$

where $\rho_{s,i}$ has the same dimensionless scale interpretation as $\rho_{d,i}$.

Functionally, the semantic-guidance module does not directly output the final convolution radius; instead, it provides complementary semantic information for radius modulation. Compared with the density-aware module, which focuses on local geometric distribution, it emphasizes correlations and differences among local semantic features. This allows the receptive field to respond to both density variation and semantic-structure complexity. The semantic-guidance branch therefore complements density-aware modeling by supplying a semantic scale factor for subsequent joint density–semantic radius prediction.

#### 3.2.3. Adaptive Convolution Module

After obtaining the density-based scale factor $\rho_{d,i}$ from the density-aware module and the semantic scale factor $\rho_{s,i}$ from the semantic-guidance module, the adaptive convolution module jointly models them to generate the final radius and construct dynamic neighborhoods for local feature aggregation. This module is the key component that enables density–semantic collaboration in DAR-Conv. To simultaneously account for geometric distribution and semantic-structure variation, we fuse the two dimensionless scale factors as follows:

(9) $$\rho_{i}=\eta_{d}\rho_{d,i}+\eta_{s}\rho_{s,i},$$

(10) $$R_{i}=R_{\mathrm{base}}\rho_{i},$$

where $\eta_{d}$ and $\eta_{s}$ are non-negative weighting coefficients satisfying $\eta_{d}+\eta_{s}=1$, which balance the contributions of density and semantics. In our implementation, $\eta_{d}$ and $\eta_{s}$ are learnable scalar fusion weights obtained by applying a two-way softmax to unconstrained parameters, rather than manually fixed constants. In this formulation, the weighted summation is performed in the normalized radius-scale domain, while the physical spatial scale is introduced only through $R_{\mathrm{base}}$. This keeps the radius prediction dimensionally consistent while preserving the intended density-driven initialization and semantics-aware modulation.

With the final radius $R_{i}$, a dynamic neighborhood is built for each point $p_{i}$:

(11) $$N_{i}=\left\{p_{j}|∥p_{j}-p_{i}∥\leq R_{i}\right\}.$$

To avoid degenerate neighborhoods caused by extreme sparsity or local sampling gaps, the support set is further constrained by minimum and maximum neighbor counts. If $|N_{i}|<K_{\min}$, the nearest points are added until $K_{\min}$ neighbors are reached; if $|N_{i}|>K_{\max}$, only the closest $K_{\max}$ neighbors are retained. After the dynamic support $N_{i}$ is obtained, DAR-Conv applies KPConv as the feature aggregation operator on this point-wise neighborhood. The kernel-point configuration, learnable kernel weights, and distance-based influence function follow the original KPConv formulation, while the support set is determined by the predicted radius $R_{i}$ rather than a fixed radius. To keep the convolution geometry consistent across different radii, the canonical kernel-point coordinates and the KPConv influence radius are normalized by $R_{\mathrm{base}}$ and then scaled by the point-wise radius $R_{i}$ during aggregation. Thus, both the neighborhood support and the kernel influence range expand or contract with $R_{i}$, whereas the learnable kernel weights are shared across points. In this way, radius-adaptation effects are directly propagated into local representation learning through KPConv aggregation.

In summary, the adaptive convolution module completes the full process from density-based and semantic scale factors to final neighborhood construction and feature aggregation, and serves as the core component for local adaptive feature extraction in DAR-Conv. By preserving the stability of the original convolution framework while adaptively redefining neighborhoods, DAR-Conv supports more stable modeling of non-uniform point clouds in complex 3D scenes.

### 3.3. Gated Adaptive Cross-Layer Fusion

Effective fusion of encoder and decoder features is critical to point-cloud semantic segmentation performance. Shallow features usually retain rich geometric details but provide relatively weak semantic representation, while deep features contain stronger semantics but often lack fine spatial structure. If cross-level fusion is implemented by direct concatenation or simple addition, information redundancy and semantic conflict may occur between different levels, which can degrade boundary recovery and feature stability. To address this issue, we design a GACF module for adaptive encoder–decoder fusion. The module takes encoder features at level *l* and decoder features at level l+1 as inputs, and uses three mechanisms—stop-gradient feature alignment, channel-wise gated fusion, and residual enhancement—to enable effective cross-level collaboration. The enhanced fused features are then fed into the current decoder stage for subsequent feature reconstruction.

#### 3.3.1. Stop-Gradient Feature Alignment

During cross-level fusion in the decoder stage, the first issue is the spatial-resolution mismatch between encoder and decoder features. Therefore, before fusion with the corresponding encoder feature, decoder features are first upsampled to align with encoder features in spatial positions. Let the encoder feature at level *l* be

(12) $$F_{l}\in R^{N_{l}\times d},$$

and the decoder feature at level l+1 be

(13) $$G_{l+1}\in R^{N_{l+1}\times d},$$

where $N_{l}$ and $N_{l+1}$ denote the numbers of points at the two levels, and *d* is the feature dimension. Since $G_{l+1}$ has lower spatial resolution than $F_{l}$, we use nearest-neighbor upsampling to map $G_{l+1}$ to the same resolution as $F_{l}$, yielding the aligned decoder feature

(14) $${\tilde{G}}_{l}=U\left(G_{l+1}\right),$$

where U(·) denotes nearest-neighbor upsampling.

In implementation, a precomputed index mapping is used so that each high-resolution point receives the feature of its nearest low-resolution point, enabling efficient feature recovery. This strategy is simple, computationally efficient, and provides explicit spatial correspondence, making it suitable for cross-level alignment in point-cloud decoding. However, relying only on direct upsampling may allow gradients from the decoder branch to directly affect cross-level fusion through the alignment path during backpropagation. This can increase optimization coupling between levels and reduce training stability, especially when shallow geometric details and deep semantic information differ significantly. To reduce this cross-level gradient interference, we introduce a stop-gradient strategy in the alignment stage by applying gradient blocking to the upsampled decoder features. Let Detach(·) denote the gradient-stopping operation. The stop-gradient-aligned decoder feature is defined as

(15) $${\hat{G}}_{l}=\mathrm{Detach}\left({\tilde{G}}_{l}\right),$$

where ${\hat{G}}_{l}$ is the aligned feature used for subsequent cross-level fusion. Under this mechanism, upsampling still participates in forward propagation to ensure spatial correspondence, while gradients through the alignment path are suppressed during backpropagation, thereby reducing interference between levels. Importantly, Detach(·) is applied only to the aligned copy used for fusion, rather than to $G_{l+1}$ itself; therefore, the main decoder path remains trainable, and only the alignment/fusion gradient path is blocked.

With this design, the stop-gradient feature alignment first matches the spatial resolution of encoder feature $F_{l}$ and decoder feature $G_{l+1}$ through nearest-neighbor upsampling, and then suppresses gradients through the alignment path to reduce cross-level optimization interference. As a result, shallow geometric information and deep semantic information can interact effectively at a unified scale, improving the stability of cross-level feature fusion.

#### 3.3.2. Channel-Wise Gated Fusion

Encoder shallow feature $F_{l}$ mainly describes local geometry, while decoder deep feature ${\hat{G}}_{l}$ carries stronger high-level semantics. Direct concatenation or simple addition may therefore introduce redundancy and semantic conflict, which harms feature recovery quality. To address this issue, GACF uses a channel-wise gating mechanism to adaptively learn feature importance from different sources and dynamically balance geometric and semantic information.

Specifically, we first normalize encoder feature $F_{l}$ and align decoder feature ${\hat{G}}_{l}$ to reduce distribution mismatch across levels. The two features are then concatenated along the channel dimension as fusion input:

(16) $$F_{c}=\mathrm{BN}\left(F_{l}\right)⊕\mathrm{BN}({\hat{G}}_{l}),$$

where BN(·) denotes batch normalization and ⊕ denotes channel concatenation. Based on the concatenated feature, a learnable mapping generates gating weights:

(17) $$M_{l}=\sigma \left(W_{g}F_{c}\right),$$

where $W_{g}$ is a learnable parameter matrix, σ(·) is the sigmoid activation, and $M_{l}$ is the point-wise channel gating response. Because the sigmoid function outputs values in [0, 1], the gate can adaptively adjust the contribution ratio of the two feature streams.

After obtaining gating weights, the fused feature is computed as

(18) $$F_{l}^{\mathrm{fused}}=M_{l}⊙F_{l}+\left(1-M_{l}\right)⊙{\hat{G}}_{l},$$

where ⊙ denotes element-wise channel multiplication. This equation shows that gate $M_{l}$ controls the relative contribution of encoder and decoder features: when $M_{l}$ is larger, more shallow geometric details are preserved; when $M_{l}$ is smaller, more deep semantic information is introduced. In this way, GACF adaptively selects fusion behavior for different levels and regions instead of using a fixed fusion rule.

Channel-wise gated fusion is therefore not a simple summation of encoder and decoder features. It controls cross-level information flow through learned gates, supports multi-level integration at a unified scale, and provides higher-quality inputs for residual enhancement and decoding.

#### 3.3.3. Residual Enhancement and Output Refinement

After channel-wise gated fusion, the fused feature $F_{l}^{\mathrm{fused}}$ already contains both shallow geometric details and deep semantic information. However, directly feeding it to the next decoder stage may still cause two issues: (1) distribution mismatch between the fused feature and the original decoder feature may disrupt feature continuity along the decoding path; and (2) newly introduced cross-level interactions may not be fully exploited without further refinement. Therefore, we introduce residual enhancement in GACF to stabilize fused features and produce output features suitable for the current decoder stage.

Based on $F_{l}^{\mathrm{fused}}$, residual information from the decoder branch is injected to preserve semantic-path continuity. Let $W_{r}$ be a linear projection matrix. The residual-enhanced output is defined as

(19) $$F_{l}^{\mathrm{out}}=\mathrm{LayerNorm}\left(F_{l}^{\mathrm{fused}}+W_{r}{\hat{G}}_{l}\right),$$

where ${\hat{G}}_{l}$ denotes the aligned decoder feature after upsampling and gradient blocking, $W_{r}{\hat{G}}_{l}$ projects decoder features into the same representation space as fused features, and LayerNorm(·) denotes layer normalization.

This design has two main benefits. First, the residual connection preserves high-level semantic information from the decoder branch during fusion and avoids excessive perturbation of original semantic representations, thus improving decoder-path stability. Second, layer normalization alleviates training instability caused by post-fusion distribution shifts and provides smoother, more consistent outputs for subsequent decoding.

The output feature $F_{l}^{\mathrm{out}}$ is then passed to subsequent decoding modules for feature reconstruction.

### 3.4. Loss Function Design

DANet is optimized with a joint objective composed of a semantic segmentation loss, a geometric consistency constraint, and a regularization term to improve classification accuracy and structural consistency in point-cloud semantic segmentation. Specifically, $L_{\mathrm{seg}}$ provides point-wise semantic supervision for accurate classification, $L_{\mathrm{geo}}$ constrains local geometric coherence to improve the stability of DAR-Conv under non-uniform sampling, and $L_{\mathrm{reg}}$ suppresses overfitting and enhances generalization. The overall loss is defined as

(20) $$L_{\mathrm{total}}=\lambda_{\mathrm{seg}}L_{\mathrm{seg}}+\lambda_{\mathrm{geo}}L_{\mathrm{geo}}+\lambda_{\mathrm{reg}}L_{\mathrm{reg}},$$

where $\lambda_{\mathrm{seg}}$, $\lambda_{\mathrm{geo}}$, and $\lambda_{\mathrm{reg}}$ are weighting coefficients for the three terms. In all experiments, we set $\lambda_{\mathrm{seg}}=1.0$, $\lambda_{\mathrm{geo}}=0.1$, and $\lambda_{\mathrm{reg}}=1\times 10^{-4}$ after validation-based tuning, and keep them fixed across datasets.

#### 3.4.1. Semantic Segmentation Loss

For point-cloud semantic segmentation, we use cross-entropy loss as the primary supervision term to measure the discrepancy between predicted class distributions and ground-truth labels at each point. Let *N* be the number of points and *C* the number of classes. The segmentation loss is

(21) $$L_{\mathrm{seg}}=-\frac{1}{N}\sum_{i=1}^{N}\sum_{c=1}^{C}y_{i,c}\log\left({\hat{y}}_{i,c}\right),$$

where $y_{i,c}$ is the ground-truth indicator of point *i* for class *c*, and ${\hat{y}}_{i,c}$ is the predicted probability.

Cross-entropy directly supervises point-wise classification and encourages discriminative semantic feature learning. For class-imbalanced data, class weights can be further introduced to alleviate dominance of frequent classes during training.

#### 3.4.2. Geometric Consistency Constraint

Because DAR-Conv adaptively modulates receptive fields during feature extraction, convolution neighborhoods vary with local density and semantic context. To avoid instability in local geometric representation during this process, we introduce a geometric consistency term that enforces smoothness within local neighborhoods.

Let $f_{p}$ denote the feature vector of point *p*, and N(p) its neighborhood set. The geometric consistency constraint is defined as

(22) $$L_{\mathrm{geo}}=\sum_{p\in P}\sum_{q\in N(p)}{∥f_{p}-f_{q}∥}_{2}^{2}\exp\left(-\frac{{∥p-q∥}_{2}^{2}}{\sigma^{2}}\right),$$

where σ is the spatial decay factor that controls the influence range within each neighborhood. In implementation, σ is set to the dataset-specific base radius $R_{\mathrm{base}}$, i.e., 0.10 m for S3DIS and 0.20 m for NPM3D, so that the decay scale is consistent with the neighborhood scale used by DAR-Conv.

This term uses Gaussian weighting to penalize feature differences in local neighborhoods, encouraging nearby points to remain consistent in feature space and thereby improving continuity and stability of point-cloud representations. For scenes with large density variation and complex structural boundaries, this constraint helps reduce local feature fluctuation and improves modeling around structural regions and semantic boundaries.

#### 3.4.3. Regularization Term

To suppress overfitting and improve generalization, we include an $L_{2}$ regularization term during training:

(23) $$L_{\mathrm{reg}}=\sum_{l=1}^{L}{∥W^{\left(l\right)}∥}_{2}^{2},$$

where $W^{\left(l\right)}$ denotes learnable parameters at layer *l*, and *L* is the total number of layers.

By constraining parameter magnitudes, this term reduces model complexity and helps limit overfitting across different scenes.

## 4. Experiments

### 4.1. Datasets

We conduct experiments on two representative point-cloud semantic segmentation benchmarks, namely S3DIS [24] and NPM3D [25], to evaluate the effectiveness of DANet in practical 3D perception settings. Specifically, S3DIS is a representative indoor benchmark with diverse scenes such as offices, meeting rooms, and corridors, making it suitable for validating local geometric modeling and semantic discrimination in structured indoor environments. NPM3D is a large-scale outdoor benchmark with complex spatial structure and substantial density variation, making it suitable for analyzing behavior under challenging outdoor acquisition conditions. By conducting experiments on these datasets, we systematically evaluate the proposed method across diverse indoor and outdoor scenes with varying density patterns and structural complexity. For dataset splits, S3DIS follows the standard Area 5 protocol, and NPM3D follows the official Paris–Lille–3D benchmark protocol [25].

### 4.2. Evaluation Metrics

Following common practice in point-cloud semantic segmentation benchmarks and prior studies [11,12,13], we report three metrics for overall evaluation on both datasets: mean Intersection over Union (mIoU), mean Accuracy (mAcc), and Overall Accuracy (OA). mIoU is the primary metric for semantic segmentation quality, mAcc reflects category-balanced recognition, and OA measures global point-wise correctness.

### 4.3. Experimental Settings

#### 4.3.1. Training Configuration

All experiments are conducted on Ubuntu 22.04 using an NVIDIA RTX 4060 GPU with 8 GB VRAM and 32 GB system RAM. The software environment is Python 3.10 with PyTorch 2.1.0. We use AdamW for optimization with an initial learning rate of 0.001. A staged learning-rate decay strategy is adopted, where the learning rate is multiplied by 0.5 every 50 epochs. The maximum number of training epochs is 500, and early stopping is applied when validation performance does not improve for 20 consecutive epochs. The batch size is set to 1, mainly due to GPU memory limitations for large point-cloud blocks; each training sample still contains a large subcloud rather than a single point. The unconstrained parameters corresponding to $\eta_{d}$ and $\eta_{s}$ are initialized equally, giving $\eta_{d}=\eta_{s}=0.5$ at the start of training, and are then learned end-to-end with the rest of the network.

#### 4.3.2. Neighborhood and Timing Settings

The base radius $R_{\mathrm{base}}$ is set according to the dataset scale: 0.10 m for S3DIS and 0.20 m for NPM3D. For all experiments, dynamic neighborhoods are constrained with $K_{\min}=8$ and $K_{\max}=64$ using the nearest-neighbor fallback and truncation rule described above. Inference latency is measured for DANet and its ablation variants in inference mode, with batch size 1 and one post-sampling evaluation subcloud per sample; the input point count is therefore the point count provided by the evaluation sampler and is kept identical across timed variants for each sampled subcloud. Each timing run excludes disk I/O, preprocessing, sampling, and data augmentation; after 20 warm-up forward passes, latency is averaged over 100 repeated forward passes with CUDA synchronization before and after each pass.

#### 4.3.3. Data Augmentation

To reduce overfitting, we applied several point-cloud data augmentation strategies during training. Specifically, the augmentation included full-angle random rotation around the vertical Z-axis with rotation angles sampled from $\left[-180^{{}^{\circ}},\;180^{{}^{\circ}}\right]$, anisotropic scaling with scaling factors sampled from [0.9, 1.1], random flipping along the horizontal X- and Y-axes with an independent probability of 0.5 for each axis, and Gaussian coordinate jittering with a standard deviation of 0.005 m. Color dropping was not used. The same preprocessing, sampling, augmentation, optimization, and early-stopping protocol was applied to the full DANet and all ablation variants to ensure a fair comparison.

### 4.4. Experimental Results and Analysis

#### 4.4.1. Overall Segmentation Results

Table 1 summarizes the overall segmentation performance of DANet and competing methods on S3DIS and NPM3D. Under this setting, DANet obtains leading results on three reported dataset–metric combinations and the second-best result on NPM3D mAcc. The strongest results appear on the outdoor NPM3D benchmark, while the remaining performance gap is mainly concentrated on S3DIS mIoU, with a smaller gap on S3DIS OA.

On S3DIS, DANet attains the highest mAcc (89.2%) among all compared methods, improving on the second-best reported entry by 3.1 percentage points. In contrast, PTv3 remains stronger on mIoU and OA, with 75.3% and 92.0% versus DANet’s 70.9% and 90.9%. This result suggests that DANet offers stronger class-balanced recognition, whereas region-overlap quality and global consistency in cluttered indoor scenes still leave room for improvement.

On NPM3D, DANet ranks first among the reported entries on mIoU and OA, reaching 93.2% and 96.3%, respectively, and ranks second on mAcc at 93.6%. Relative to the strongest competing results, DANet improves mIoU and OA by 1.1 and 0.9 percentage points, while remaining only 0.5 points below the best mAcc. The mIoU gain suggests fewer region-level false positives and false negatives across NPM3D classes, while the OA gain shows that a larger proportion of all evaluated outdoor points are assigned correct labels.

Taken together, Table 1 shows that DANet performs especially well on the outdoor benchmark while maintaining strong class-balanced performance on indoor scenes. The remaining S3DIS gap, especially in mIoU, suggests that further gains are still possible for indoor object-level segmentation.

#### 4.4.2. Per-Class mIoU Results on S3DIS

As shown in Table 2, DANet achieves the best class-wise IoU among the compared methods on Window (71.2%) and Chair (88.0%), and is tied for the best result on Wall (85.5%) and Board (68.7%). It also ranks second on Ceiling (94.9%) and is tied for second on Sofa (68.8%). The largest gain is observed on Chair, where DANet improves on the second-best method by 4.3 points and exceeds PTv3 by 17.8 points. By contrast, the lower scores on Beam, Column, and Bookcase indicate that thin elongated structures and some long-tail indoor categories remain more challenging.

Across baselines, class-wise behavior is clearly category-dependent. Point-ASNL [20] and PTv3 [17] share the best Ceiling score (95.3%), while PA-Net [26] achieves the best Floor result (98.1%). KPConv [12] performs best on Bookcase (71.7%) and obtains high scores on Door (77.3%) and Sofa (68.8%). PointNeXt [16] leads on Table (77.6%) and is tied for the best Sofa score (71.2%), whereas PTv3 provides the strongest competing profile overall by leading on Beam (65.8%), Column (60.3%), Door (80.1%), and Clutter (67.5%), and being tied for the best results on Ceiling, Wall, Sofa, and Board. In contrast, DANet performs best on Window and Chair and is tied for the best results on Wall and Board.

Across the 13 S3DIS categories, DANet achieves best-or-tied-best results on four categories and second-or-tied-second results on two additional categories. Its lowest-class IoU is 51.0% on Bookcase, while the high-rank categories remain concentrated on structural planes and furniture classes. The remaining gaps on Beam, Column, Door, Table, Bookcase, and Clutter point to thin structures and long-tail indoor categories as the main areas for further optimization.

The relatively lower performance on Beam, Column, and Bookcase is mainly associated with data distribution and geometric ambiguity in indoor scenes. Although mAcc is computed in a class-wise manner and therefore does not simply reflect majority-class dominance, mIoU is more sensitive to false positives and false negatives around object boundaries. Beam and Column are typically thin, elongated, and under-represented structures that are spatially adjacent to dominant planar categories such as Wall, Ceiling, and Floor. When the local point density is low or the object boundary is partially occluded, the adaptive receptive field in DAR-Conv may include surrounding background points from these dominant classes, introducing contextual noise and weakening boundary discrimination. Bookcase is also challenging because it often contains heterogeneous internal objects and shares similar local geometry with Clutter, Wall, and furniture categories. Therefore, the lower IoU on these categories is likely caused by false-positive predictions and boundary leakage rather than a complete failure of class recognition. This limitation suggests that boundary-aware constraints or class-aware radius regularization may further improve performance on thin and under-represented indoor structures.

#### 4.4.3. Per-Class mIoU Results on NPM3D

As shown in Table 3, DANet achieves the best class-wise IoU on five of the eight categories, namely Building (99.0%), Bollard (93.7%), Trash Can (97.6%), Barrier (92.7%), and Pedestrian (94.0%), and ranks second on Pole (76.4%). The largest margins over the second-best methods appear on sparse foreground classes, including Barrier (+8.7), Bollard (+6.5), Pedestrian (+6.5), and Trash Can (+5.8). Based on the listed NPM3D categories in Table 3, these gains suggest that DANet is particularly effective for small-object and long-tail category modeling in urban outdoor scenes.

From a cross-method perspective, clear category preferences remain across baselines. KPConv [12] and RandLA-Net [13] are strongest on the dominant Ground category (both 99.5%), RandLA-Net achieves the highest Car score (95.3%), and MS-RRFSegNet [27] performs best on Pole (79.7%). PTv3 [17] is the strongest competing method on several sparse foreground categories, ranking second on Bollard (87.2%), Trash Can (91.8%), Barrier (84.0%), and Pedestrian (87.5%). In contrast, DANet provides the strongest overall balance between major structures and minority obstacles, with clear advantages on under-represented obstacle categories.

Across the eight NPM3D categories, DANet achieves best-or-second-best results on six categories. Its lowest-class IoU is 76.4% on Pole, while all remaining categories exceed 92%. This profile indicates that the proposed density- and semantics-adaptive receptive field is especially beneficial for compact or sparsely distributed foreground objects, where fixed neighborhoods may either miss useful contextual points or introduce excessive background interference. The strong scores on Bollard, Trash Can, Barrier, and Pedestrian are consistent with the design motivation of adapting local support according to point density and semantic cues.

For NPM3D, DANet achieves strong performance on several sparse or small foreground categories, such as Bollard, Trash Can, Barrier, and Pedestrian, indicating that the adaptive radius mechanism is effective for density-varying outdoor scenes. The improvements on these categories suggest that DAR-Conv can provide broader or more suitable contextual support when local neighborhoods are sparse or structurally complex. However, DANet obtains a slightly lower IoU on the Ground class than KPConv and RandLA-Net. Since Ground is already a highly saturated and dense planar category, this decrease is mainly attributed to boundary-level errors rather than failures on large homogeneous ground surfaces. In boundary regions between Ground and adjacent objects, such as curbs, poles, bollards, barriers, pedestrians, vehicles, vegetation, and building facades, mixed-category neighborhoods and abrupt density changes may reduce planar continuity. A similar effect can also be observed for Pole and Car, whose performance is affected by thin structures, occlusion, local density variation, and semantic confusion with surrounding objects. These results suggest that DAR-Conv improves the discrimination of sparse foreground objects and complex boundaries, while a small trade-off may occur on already saturated dense planar regions.

### 4.5. Ablation Study

This subsection evaluates the contribution of the main DANet components through ablation experiments. In the ablation notation, A, B, C, and D denote the GACF module, the density-aware branch, the semantic-guidance branch, and the complete DAR-Conv local operator, respectively. The four ablation variants are defined as follows.

The w/o A variant removes GACF and uses a plain skip fusion based on nearest-neighbor upsampling, encoder–decoder feature concatenation, and decoder projection. The w/o B variant removes the density-aware branch, so the adaptive radius is computed only from the semantic scale, i.e., $\rho_{i}=\rho_{s,i}$ and $R_{i}=R_{\mathrm{base}}\rho_{s,i}$. The w/o C variant removes the semantic-guidance branch and uses only the density scale, i.e., $\rho_{i}=\rho_{d,i}$ and $R_{i}=R_{\mathrm{base}}\rho_{d,i}$. The w/o D variant replaces DAR-Conv with a fixed-radius KPConv operator. The detailed experimental results are reported in Table 4.

Compared with the full DANet (mIoU/mAcc = 93.2/93.6), w/o D produces the largest performance drop (ΔmIoU = −6.9, ΔmAcc = −4.0), indicating that the adaptive local operator is the most influential component in the tested setting. The w/o B variant gives the second-largest degradation (ΔmIoU = −2.8, ΔmAcc = −2.4), showing the importance of density-aware radius prediction for non-uniform point clouds. Removing GACF also reduces performance (w/o A; ΔmIoU = −1.1, ΔmAcc = −0.8), which confirms the benefit of adaptive encoder–decoder feature fusion. The w/o C variant causes a smaller but consistent decline (ΔmIoU = −0.7, ΔmAcc = −0.6), suggesting that semantic guidance further refines local receptive-field adaptation.

The ablated variants have slightly lower inference latency than the full model, reflecting their reduced fusion or aggregation complexity. However, all simplified variants also show lower mIoU and mAcc, which indicates that the accuracy gain of the full model is not obtained from a single module but from the combined effect of DAR-Conv, density-aware radius prediction, semantic guidance, and GACF.

## 5. Conclusions

DANet addresses 3D point-cloud semantic segmentation through joint density- and semantics-adaptive convolution. Its DAR-Conv operator adjusts local receptive fields according to point density and semantic cues, improving feature extraction under the non-uniform sampling patterns commonly observed in LiDAR and depth-camera data. The GACF module further strengthens encoder–decoder interaction through alignment-aware fusion, gated feature selection, and residual refinement, which improves multi-scale semantic consistency. On S3DIS and NPM3D, DANet shows high mAcc on S3DIS and stronger mIoU/OA performance on NPM3D, although S3DIS mIoU and OA still leave room for improvement. The experiments suggest that density- and semantics-aware receptive-field adaptation is useful under the tested settings, while broader validation on additional datasets and deployment scenarios remains necessary.

DANet is not expected to be universally optimal in all scenarios. DAR-Conv is most beneficial when point density varies substantially or when semantic boundaries require flexible contextual support. In nearly uniform point clouds, adaptive radius prediction may add only limited benefit. Similar limitations may appear in extremely sparse scans with unreliable local statistics or in scenes with weak semantic cues. For resource-constrained applications, the adaptive mechanism may also introduce extra overhead when latency is more critical than accuracy. These limitations suggest that the base radius, density–semantic weighting, and fusion strategy may still require dataset- or application-specific tuning. Future work will focus on improving computational efficiency, increasing stability under extreme sparsity and weak semantic guidance, and extending the framework to multimodal and temporal point-cloud understanding tasks.

## Figures and Tables

**Table 1:** Overall segmentation performance on S3DIS and NPM3D.

Dataset	Method	mIoU	mAcc	OA
S3DIS	KPConv [12]	69.6	84.3	85.7
	PA-Net [26]	59.9	82.5	84.4
	RandLA-Net [13]	70.0	82.0	88.0
	LCPFormer [18]	70.2	76.8	90.8
	PTv1 [15]	70.0	76.8	90.4
	PTv3 [17]	**75.3**	86.1	**92.0**
	SCF-Net [19]	71.6	82.7	88.4
	Ours	70.9	**89.2**	90.9
NPM3D	KPConv [12]	80.9	87.3	93.5
	PA-Net [26]	92.1	**94.1**	95.4
	RandLA-Net [13]	76.8	92.7	94.2
	PTv3 [17]	88.5	90.8	94.2
	Ours	**93.2**	93.6	**96.3**

**Bold**: best result; underlined: second best result.

**Table 2:** Per-class mIoU (%) on S3DIS.

Methods	Ceiling	Floor	Wall	Beam	Column	Window	Door	Table	Chair	Sofa	Book Case	Board	Clutter
Point-ASNL [20]	**95.3**	97.9	81.9	47.0	48.0	67.3	70.5	71.3	77.8	50.7	60.4	63.6	62.8
KPConv [12]	93.7	92.0	82.5	62.5	49.5	65.7	77.3	57.8	64.0	68.8	**71.7**	60.1	59.6
PA-Net [26]	93.2	**98.1**	80.6	13.3	31.1	32.8	56.7	75.1	83.7	38.9	69.9	52.4	53.0
PointNeXt [16]	94.3	97.5	84.7	55.6	58.1	66.1	78.2	**77.6**	74.1	**71.2**	67.3	65.7	64.8
SCF-Net [19]	93.3	96.4	80.9	64.9	47.4	64.5	70.1	71.4	81.6	67.2	64.4	67.5	60.9
PTv3 [17]	**95.3**	95.3	**85.5**	**65.8**	**60.3**	71.1	**80.1**	77.3	70.2	**71.2**	70.2	**68.7**	**67.5**
Ours	94.9	92.0	**85.5**	57.5	53.3	**71.2**	69.0	67.0	**88.0**	68.8	51.0	**68.7**	54.8

**Bold**: best result; underlined: second best result.

**Table 3:** Per-class mIoU (%) on NPM3D.

Methods	Ground	Building	Pole	Bollard	Trash Can	Barrier	Pedestrian	Car
MS-RRFSegNet [27]	98.6	98.0	**79.7**	74.3	75.1	57.9	55.9	82.0
KPConv [12]	**99.5**	94.0	71.3	83.1	78.7	47.7	78.2	94.4
RandLA-Net [13]	**99.5**	97.0	71.0	86.7	50.5	65.5	49.1	**95.3**
PTv3 [17]	99.1	97.3	70.5	87.2	91.8	84.0	87.5	90.9
Ours	99.0	**99.0**	76.4	**93.7**	**97.6**	**92.7**	**94.0**	93.6

**Bold**: best result; underlined: second best result.

**Table 4:** Ablation study results for DANet.

Method	mIoU (%)	mAcc (%)	Training Time (h)	Latency (ms/Sample)
Full Model	93.2	93.6	25.4	45.2
w/o A	92.1	92.8	23.8	43.8
w/o B	90.4	91.2	23.9	43.9
w/o C	92.5	93.0	24.7	44.7
w/o D	86.3	89.6	23.5	43.2

## Data Availability

The S3DIS and NPM3D datasets used in this study are publicly available through their official dataset webpages or repositories.

## Abbreviations

The following abbreviations are used in this manuscript:

DAR-Conv	Density-Adaptive Radius Convolution
GACF	Gated Adaptive Cross-Layer Fusion
mIoU	Mean Intersection over Union
mAcc	Mean Accuracy
OA	Overall Accuracy

## References

[1] Zhang R., Wu Y., Jin W., Meng X. (2023). Deep-Learning-Based Point Cloud Semantic Segmentation: A Survey. Electronics.

[2] Dumić E., da Silva Cruz L.A. (2025). Three-Dimensional Point Cloud Applications, Datasets, and Compression Methodologies for Remote Sensing: A Meta-Survey. Sensors.

[3] Han T., Chen Y., Ma J., Liu X., Zhang W., Zhang X., Wang H. (2024). Point cloud semantic segmentation with adaptive spatial structure graph transformer. Int. J. Appl. Earth Obs. Geoinf..

[4] Sun Y., Zhang X., Miao Y. (2024). A review of point cloud segmentation for understanding 3D indoor scenes. Vis. Intell..

[5] Zhou W., Liu K., Jin W., Wang Q., She Y., Yu Y., Ma C. (2025). Advancements in deep learning for point cloud classification and segmentation: A comprehensive review. Comput. Graph..

[6] Betsas T., Georgopoulos A., Doulamis A., Grussenmeyer P. (2025). Deep Learning on 3D Semantic Segmentation: A Detailed Review. Remote Sens..

[7] Milioto A., Vizzo I., Behley J., Stachniss C. RangeNet++: Fast and Accurate LiDAR Semantic Segmentation. Proceedings of the IEEE/RSJ International Conference on Intelligent Robots and Systems (IROS).

[8] Puy G., Boulch A., Marlet R. Using a Waffle Iron for Automotive Point Cloud Semantic Segmentation. Proceedings of the IEEE/CVF International Conference on Computer Vision (ICCV).

[9] Zhu X., Zhou H., Wang T., Hong F., Ma Y., Li W., Li H., Lin D. Cylindrical and Asymmetrical 3D Convolution Networks for LiDAR Segmentation. Proceedings of the IEEE/CVF Conference on Computer Vision and Pattern Recognition (CVPR).

[10] Kolodiazhnyi M., Vorontsova A., Konushin A., Rukhovich D. OneFormer3D: One Transformer for Unified Point Cloud Segmentation. Proceedings of the IEEE/CVF Conference on Computer Vision and Pattern Recognition (CVPR).

[11] Qi C.R., Yi L., Su H., Guibas L.J. (2017). PointNet++: Deep Hierarchical Feature Learning on Point Sets in a Metric Space. Adv. Neural Inf. Process. Syst..

[12] Thomas H., Qi C.R., Deschaud J.E., Marcotegui B., Goulette F., Guibas L.J. KPConv: Flexible and Deformable Convolution for Point Clouds. Proceedings of the IEEE/CVF International Conference on Computer Vision (ICCV).

[13] Hu Q., Yang B., Xie L., Rosa S., Guo Y., Wang Z., Trigoni N., Markham A. (2022). Learning Semantic Segmentation of Large-Scale Point Clouds with Random Sampling. IEEE Trans. Pattern Anal. Mach. Intell..

[14] Landrieu L., Simonovsky M. Large-Scale Point Cloud Semantic Segmentation with Superpoint Graphs. Proceedings of the IEEE Conference on Computer Vision and Pattern Recognition (CVPR).

[15] Zhao H., Jiang L., Jia J., Torr P.H.S., Koltun V. Point Transformer. Proceedings of the IEEE/CVF International Conference on Computer Vision (ICCV).

[16] Qian G., Li Y., Peng H., Mai J., Hammoud H.A.A.K., Elhoseiny M., Ghanem B. PointNeXt: Revisiting PointNet++ with Improved Training and Scaling Strategies. Proceedings of the Advances in Neural Information Processing Systems.

[17] Wu X., Jiang L., Wang P.S., Liu Z., Liu X., Qiao Y., Ouyang W., He T., Zhao H. Point Transformer V3: Simpler Faster Stronger. Proceedings of the IEEE/CVF Conference on Computer Vision and Pattern Recognition (CVPR).

[18] Huang Z., Zhao Z., Li B., Han J. (2023). LCPFormer: Towards Effective 3D Point Cloud Analysis via Local Context Propagation in Transformers. IEEE Trans. Circuits Syst. Video Technol..

[19] Fan S., Dong Q., Zhu F., Lv Y., Ye P., Wang F.Y. SCF-Net: Learning Spatial Contextual Features for Large-Scale Point Cloud Segmentation. Proceedings of the IEEE/CVF Conference on Computer Vision and Pattern Recognition (CVPR).

[20] Yan X., Zheng C., Li Z., Wang S., Cui S. PointASNL: Robust Point Clouds Processing Using Nonlocal Neural Networks with Adaptive Sampling. Proceedings of the IEEE/CVF Conference on Computer Vision and Pattern Recognition (CVPR).

[21] Xu M., Ding R., Zhao H., Qi X. PAConv: Position Adaptive Convolution With Dynamic Kernel Assembling on Point Clouds. Proceedings of the IEEE/CVF Conference on Computer Vision and Pattern Recognition (CVPR).

[22] Wu X., Lao Y., Jiang L., Liu X., Zhao H. (2022). Point Transformer V2: Grouped Vector Attention and Partition-Based Pooling. Adv. Neural Inf. Process. Syst..

[23] Wang Z., Wang Y., An L., Liu J., Liu H. (2022). Local Transformer Network on 3D Point Cloud Semantic Segmentation. Information.

[24] Armeni I., Sener O., Zamir A.R., Jiang H., Brilakis I., Fischer M., Savarese S. 3D Semantic Parsing of Large-Scale Indoor Spaces. Proceedings of the IEEE Conference on Computer Vision and Pattern Recognition (CVPR).

[25] Roynard X., Deschaud J.E., Goulette F. (2018). Paris-Lille-3D: A Large and High-Quality Ground-Truth Urban Point Cloud Dataset for Automatic Segmentation and Classification. Int. J. Robot. Res..

[26] Ren D., Wu Z., Li J., Yu P., Guo J., Wei M., Guo Y. (2022). Point attention network for point cloud semantic segmentation. Sci. China Inf. Sci..

[27] Luo H., Chen C., Fang L., Khoshelham K., Shen G. (2020). MS-RRFSegNet: Multiscale Regional Relation Feature Segmentation Network for Semantic Segmentation of Urban Scene Point Clouds. IEEE Trans. Geosci. Remote. Sens..

